# Long-term bidirectional association between asthma and attention deficit hyperactivity disorder: A big data cohort study

**DOI:** 10.3389/fpsyt.2022.1044742

**Published:** 2023-01-19

**Authors:** Hye Jin Park, Young Hyeon Kim, Da Ye Na, Seong Won Jeong, Min Gyu Lee, Jong-Hwan Lee, Yun Na Yang, Min Gu Kang, Sang Woo Yeom, Jong Seung Kim

**Affiliations:** ^1^Department of Medical Informatics, Jeonbuk National University Medical School, Jeonju, Republic of Korea; ^2^Department of Otorhinolaryngology, Jeonbuk National University Medical School, Jeonju, Republic of Korea; ^3^Research Institute of Clinical Medicine of Jeonbuk National University—Biomedical Research Institute of Jeonbuk National University Hospital, Jeonju, Republic of Korea

**Keywords:** asthma, ADHD, National Health Insurance Service (NHIS), cohort study, psychiatry

## Abstract

**Background:**

Previous studies have argued that attention deficit hyperactivity disorder (ADHD) is associated with asthma. However, reliable evidence to verify this association has not yet been reported.

**Objectives:**

To investigate the bidirectional association between asthma and ADHD through a 12-year big data cohort study.

**Methods:**

The independent variable group was extracted from 3.5 million individuals randomly sampled by the National Health Insurance Service (NHIS). In Study 1, the incidence of ADHD according to asthma was evaluated, while in Study 2, the incidence of asthma according to ADHD was analyzed. Propensity score (PS) matching with several variables was used to obtain a control group.

**Measurements and main results:**

In Study 1, the asthma group included 131,937 individuals and the non-asthma group included 131,937 individuals. The adjusted hazard ratio (aHR) for ADHD in the asthma group was 1.17 [95% confidence interval (CI): 1.11–1.23]. In subgroup analysis, the aHRs for ADHD of individuals in the subgroups male sex, 0–5 years old, 6–10 years old, atopic dermatitis, allergic rhinitis, Charlson comorbidity index (CCI) 1, and CCI > 2 were significant (aHR: 2.83, 1.70, 1.79, 1.09, 1.15, 1.06, and 1.49, respectively). In Study 2, ADHD was found to significantly affect asthma in all age groups (aHRs of the subgroups 0∼60 and 0∼17 years old were 1.10 and 1.09, respectively). In the 0∼17 years old subgroup, the association of ADHD with asthma was greater with younger age (aHRs of the subgroups 0∼5 and 6∼10 years old were 2.53 and 1.54, respectively).

**Conclusion:**

From long-term follow-up, the incidence of ADHD was 1.17 times higher in the asthma group than in the control group. The incidence of asthma was 1.10 times higher in the ADHD group than in the control group. Asthma and ADHD have a bidirectional relationship, and childhood asthma and ADHD should be rigorously managed.

## Introduction

Attention deficit hyperactivity disorder (ADHD) is a disease characterized by continuous inattention or hyperactivity and impulsivity that hinder functioning and development (1). The prevalence of ADHD in childhood is estimated to be 5.9%, and the prevalence of ADHD in adulthood is estimated to be 4.4% (2). Individuals suffering from ADHD have difficulties with attention, cognition, social interaction, and academic performance (3). ADHD has several comorbidities, such as anxiety, bipolar disorder, depression, drug or alcohol abuse, and antisocial disorder (4). Asthma is an allergic disease with a pervasive prevalence. Because the bronchi in the lungs become sensitive, they become increasingly narrow, causing the patient to cough excessively and experience shortness of breath. In asthma, in addition to the three major symptoms of cough, dyspnea, and wheezing, there are minor symptoms such as limitations in everyday activities, which need to be differentiated from mental disorders such as ADHD (5).

Previous studies on the association between ADHD and allergic diseases have primarily been conducted in children. However, for both ADHD and allergic diseases, an increasing trend is observed in all age groups, including children (6). In a survey of diagnostic trends in adult ADHD in California from January 1, 2007 to December 31, 2016, an annual increase was confirmed in all races/ethnic groups (6). Asthma is a clinical syndrome affecting individuals of all ages, and the prevalence of asthma worldwide has increased in the last century (7).

Asthma patients have a high prevalence of psychological disorders (8, 9). The association of asthma with multiple psychiatric disorders was reported in a study on 18–79-year-old individuals from a German population using a self-reported questionnaire (8). Based on telephone interviews with adolescents aged 11–17 with asthma and controls, those with asthma were approximately twice as likely to experience anxiety and depression disorders as those without asthma (9). Specifically, there are recent reports that asthma is genetically associated with ADHD in several mental disorders (10, 11). A large-scale study on monozygotic twins reported that the risk of ADHD was higher in the asthma group than that in the non-asthma group, suggesting a genetic correlation between asthma and ADHD (12). In a study using the UK Biobank, there was evidence of a strong genetic association between asthma and ADHD at the genome-wide level (13). A National Survey of Children’s Health data showed that ADHD was twice as common in the asthma group and three times more common in the severe asthma group (14).

On the other hand, an observational study reported that asthma and ADHD were inherited independently (15). Another study reported independent transmission of ADHD and asthma in proband groups, concluding that the symptoms of ADHD and asthma should be clearly distinguished and treated separately (16).

As previous studies on the association between ADHD and asthma have not provided consistent results (8–16), this study aimed to investigate the bidirectional association between asthma and ADHD through a 12-year big data cohort study provided by the National Health Insurance Service (NHIS).

## Materials and methods

### Database and type of study

As the NHIS is a single-payer government-run healthcare system operated by the Ministry of Health and Welfare, data from the entire population of South Korea can be collected for insurance claims. In addition, private hospitals are not allowed to withdraw from National Health Insurance, so data from all citizens receiving medical treatment in South Korea are recorded by the NHIS (17).

The National Health Insurance Service—National Sample Cohort consists of an approximately 12-year medical history of 3.5 million individuals, 7% of the total population of South Korea, whose data on sex, age, health insurance premium, and residential area were randomly sampled from the NHIS database. This retrospective cohort study included fundamental demographic variables, such as patient’s sex and age, and medical information, such as the patient’s diagnosis code, treatment history, medication, insurance claims, and hospital visit dates. Asthma and ADHD were classified according to the presence or absence of diagnosis.

We conducted a bidirectional study on asthma and ADHD.

### Patient selection

Based on the International Classification of Diseases, 10th revision diagnosis code (ICD-10 Code), the asthma group was those diagnosed with J45 or J46 twice within 90 days between 2009 and 2010. The ADHD group was those diagnosed with either F90 or F988 twice within 90 days during the same period.

### Study design

#### Study 1

**FIGURE 1:**
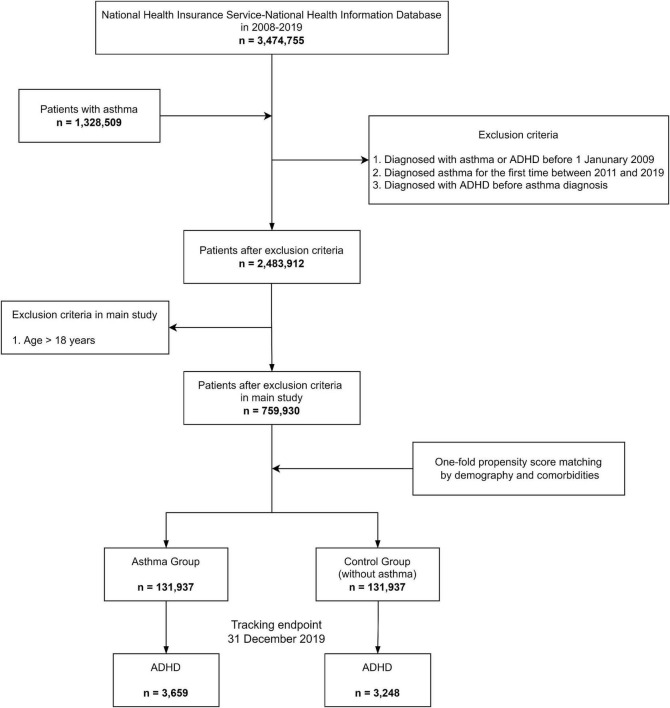
Study 1. Flow chart for experiment design.

In Study 1, we investigated the relationship between asthma as an independent variable and ADHD as a dependent variable. Since the NHIS database provided medical history between 2008 and 2019, we included a 1-year (2008) wash-out period to avoid misinterpreting the order of occurrence between the two diseases. The study was performed in three age ranges: (1) 0–17 years old, (2) 18–60 years old, and (3) 0–60 years old, and our main study group included individuals aged 0–17 years. There were three exclusion criteria: (1) patients with asthma and ADHD with an initial diagnosis before January 1, 2009 during the wash-out period; (2) patients diagnosed with asthma for the first time during the period 2011–2019; and (3) patients with at least one record of ADHD diagnosed earlier or at the same time as the asthma diagnosis. From the asthma cohort (defined earlier), the control group was matched by 1:1 propensity score (PS) matching considering seven covariates, including the Charlson comorbidity index (CCI). We conducted PS matching using a “greedy nearest neighbor” algorithm with a 1:1 ratio (18–24). The success of PS matching was confirmed by standardized mean differences (SMDs) in each group (Table 1) (18–24). The initiation point for the study group was the date of the first asthma diagnosis, and that for the control group was the date of the first visit to the hospital from January 1, 2009. The study and control groups were followed up until December 31, 2019, tracking the development of ADHD (Figure 1).

**TABLE 1:** Demography of asthma and non-asthma groups (Study 1).

Variable	Study (asthma) group (*n* = 131,937)	Control (non-asthma) group (*n* = 131,937)	SMD
Sex			0.01
Female	60,464	60,972	
Male	71,473	70,965	
Age, years	6.25 (4.43)	6.40 (4.52)	0.06
0–5	65,587	65,057	
6–10	42,074	39,711	
11–17	24,276	27,169	
Economic status	12.47 (5.62)	12.40 (5.53)	0.05
<20%	16,537	15,850	
20–80%	76,657	79,740	
>80%	38,743	36,347	
Region			0.06
Rural	42,755	39,017	
Metro	89,182	92,920	
Atopic dermatitis			0.10
Yes	29,198	23,834	
No	102,739	108,103	
Allergic rhinitis			0.03
Yes	5,955	5,201	
No	125,982	126,736	
CCI	0.43 (0.56)	0.38 (0.54)	0.09
0	78,764	83,901	
1	50,389	46,109	
2	2,784	1,927	

SMD, standardized mean difference and CCI, Charlson comorbidity index.

#### Sensitivity analyses

To enhance the robustness of the results in our main study, we also performed four sensitivity analyses, which showed that the results did not change based on the following: (1) operational definitions for asthma (diagnosis twice within 90 days, and within 30 days) and ADHD (diagnosis twice within 90 days, and diagnosis once); (2) matching variables (before matching, minimal matching (age and sex), demographic matching (age, sex, residential area, and economic status), full matching (age, sex, residential area, economic status, atopic dermatitis, allergic rhinitis, and CCI); (3) recruitment periods for the asthma group (4, 3, 2 years); and (4) matching ratios (1:3, 1:2, 1:1).

#### Study 2

Study 2 investigated the reciprocal of the relationship in Study 1, that is, the relationship between ADHD as an independent variable and asthma as a dependent variable. The criteria for Study 2 were the reverse of those of Study 1. In addition, Study 2 was performed in three age ranges: (1) 0–17 years old, (2) 18–60 years old, and (3) 0–60 years old. The experimental cohort was defined as patients diagnosed with ADHD between January 1, 2009 and December 31, 2010. This time, target disease was defined as a diagnosis of asthma between 2009 and 2019. The time order between the occurrence of asthma and ADHD was the same as that in Study 1, and a 1-year wash-out period was included. There were also three exclusion criteria: (1) patients with asthma and ADHD with an initial diagnosis before January 1, 2009 during the wash-out period; (2) patients diagnosed with ADHD for the first time during the period 2011–2019; and (3) patients with at least one record of asthma diagnosis earlier or at the same time as ADHD diagnosis.

#### Covariate conditions

The covariates included were as follows: (1) age (0–5, 6–10, and 11–17 years), (2) residential area (metropolitan and rural), (3) economic status divided by health insurance premium (the insurance premium based on income was divided into 20 deciles) (top 20, 20–80, and bottom 20%), and baseline comorbidities with CCI. The individual comorbidities included atopic dermatitis (ICD-10 code L20; diagnosed twice within 90 days) and allergic rhinitis (ICD-10 code J30 with Korean treatment codes E7151, E7152, EY853, and EY854). For the CCI, comorbidities considered with weighting were as follows: (1) Acute myocardial infarction (ICD-10 codes I21, I22, and I25), (2) Diabetes mellitus (ICD-10 codes E101, E109, E111, E119, E130, E131, E140, and E141), (3) Complications from diabetes mellitus (ICD-10 codes E102, E104, E112, E114, E132, E134, E142, and E144), (4) Congestive heart failure (ICD-10 code I50), (5) Peripheral vascular disease (ICD-10 codes I70-I79), (6) Cerebrovascular disease (ICD-10 codes I60-I69), (7) Dementia (ICD-10 codes F03, and G30), (8) Chronic obstructive pulmonary disease (ICD-10 codes J41-J45, J47, and J64), (9) Connective tissue disease (ICD-10 codes M30-M36, and M06), (10) Peptic ulcer (ICD-10 codes K25-K26), (11) Liver disease (ICD-10 codes B18, B19, and K70-K77), (12) Hemiplegia (ICD-10 codes G80-G82), (13) Renal kidney failure (ICD-10 codes N17-N19), (14) Cancer (ICD-10 codes C0, C3, C5, C6, C43, C45-C49, C70-C72, C74, C75, and C81-C96), (15) Cancer without specification of site (ICD-10 codes C76-C80), (16) Acquired immune deficiency syndrome (ICD-10 codes B20-B24).

#### Statistical analysis

The major outcome was the occurrence of ADHD and asthma in Studies 1 and 2, respectively. The hazard ratio (HR) in the Cox proportional hazards model was calculated considering the time variable (between the end and start points of the study period). When considering the relationship between one independent variable and the dependent variable, the adjusted HR (aHR) was obtained by considering all other variables, whereas the unadjusted HR was calculated without considering other variables. The cumulative HR was obtained through Kaplan–Meier survival analysis, and the R 3.5.3 statistical program (R Foundation for Statistical Computing, Vienna, Austria) was used to analyze the results.

## Results

### Study 1

Study 1 investigated the relationship between asthma as an independent variable and ADHD as a dependent variable. There were 131,937 participants in the asthma group and the same number in the PS-matched non-asthma control group. These groups were well-distributed with regard to sex, age, economic status, residential area, atopic dermatitis, allergic rhinitis, and CCI (Table 1). All SMDs were less than or equal to 0.1, indicating that they were well-balanced.

**FIGURE 2:**
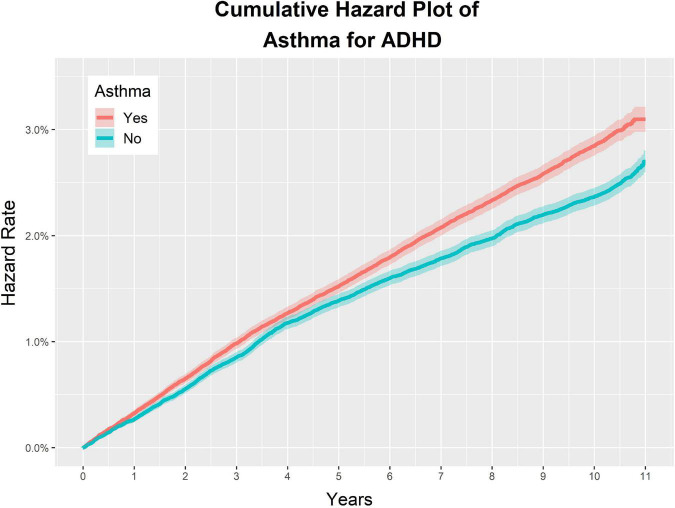
Study 1. Overall cumulative hazard rate for ADHD in the asthma group and the non-asthma group.

**FIGURE 3:**
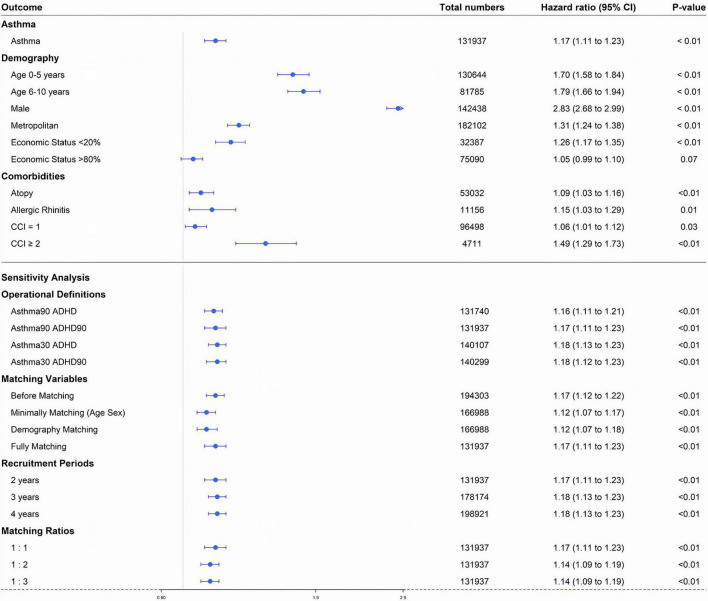
Study 1. Forest plot of adjusted hazard ratio for each factor in a multivariate Cox proportional hazard model: asthma, sex, economic status, age, residential area, underlying disease (atopic dermatitis, allergic rhinitis), and Charlson comorbidity index. CCI, Charlson comorbidity index. Forest plot of adjusted hazard ratio of asthma factor through sensitivity analyses Asthma90 ADHD, asthma defined by two diagnoses within 90 days and ADHD defined by one diagnosis; Asthma90 ADHD90, asthma defined by two diagnoses within 90 days and ADHD defined by two diagnoses within 90 days; Asthma30 ADHD, asthma defined by two diagnoses within 30 days and ADHD defined by one diagnosis; Asthma30 ADHD90, asthma defined by two diagnoses within 30 days and ADHD defined by two diagnoses within 90 days.

The incidence of ADHD per 10,000 person-years (PY) was 2.90 in the asthma group, and 2.44 in the non-asthma control group. The adjusted HR (aHR) for the asthma experimental group and the control group for ADHD was 1.17 (95% CI: 1.11–1.23), indicating that the incidence of ADHD in the asthma group was 1.17 times higher than that in the control group. The univariate crude HR of the two cohorts for ADHD was 1.20 (95% CI: 1.14–1.25) (Figures 2, 3 and Table 2).

**TABLE 2:** Incidence rate and adjusted and unadjusted HRs for each group (Study 1).

Variable	Total	Cases	10,000 PY	Adjusted hazard ratio	Unadjusted hazard ratio
Total	263,874	6,907			
**Asthma**					
No	131,937	3,248	2.44	1	1
Yes	131,937	3,659	2.90	1.17 (1.11–1.23)	1.20 (1.14–1.25)
**Sex**					
Female	121,436	1,637	1.35	1	1
Male	142,438	5,270	3.81	2.83 (2.68–2.99)	2.81 (2.66–2.97)
**Age, years**					
11–17	51,445	901	1.79	1	1
6∼10	81,785	2,476	3.07	1.79 (1.66–1.94)	1.72 (1.59–1.86)
0–5	130,644	3,530	2.75	1.70 (1.58–1.84)	1.54 (1.43–1.66)
**Economic status**					
20–80%	156,397	3,926	2.55	1	1
<20%	32,387	990	3.12	1.26 (1.17–1.35)	1.22 (1.14–1.31)
>80%	75,090	1,991	2.71	1.05 (0.99–1.10)	1.06 (1.01–1.12)
**Region**					
Rural	81,772	1,807	2.24	1	1
Metro	182,102	5,100	2.86	1.31 (1.24–1.38)	1.27 (1.21–1.34)
**Atopic dermatitis**					
No	210,842	5,360	2.59	1	1
Yes	53,032	1,547	2.94	1.09 (1.03–1.16)	1.13 (1.07–1.20)
**Allergic rhinitis**					
No	252,718	6,564	2.65	1	1
Yes	11,156	343	3.12	1.15 (1.03–1.29)	1.18 (1.06–1.31)
**CCI**					
0	162,665	4,165	2.61	1	1
1	96,498	2,559	2.69	1.06 (1.01–1.12)	1.03 (0.98–1.08)
2	4,711	183	4.00	1.49 (1.29–1.73)	1.53 (1.32–1.77)

10,000 PY, per 10,000 person-years and CCI, Charlson comorbidity index.

In the asthma and matched non-asthma groups, the characteristics associated with an increased incidence of ADHD were as follows: atopic dermatitis, allergic rhinitis, CCI = 1, and CCI > 2. The aHRs of the above characteristics were 1.09 (95% CI: 1.03–1.16), 1.15 (95% CI: 1.03–1.29), 1.06 (95% CI: 1.01–1.12), and 1.49 (95% CI: 1.29–1.73), respectively (Figure 3 and Table 2).

For the demographic factors, the aHR values were as follows: (1) male sex: 2.83 (95% CI: 2.68–2.99); (2) lower 20% of insurance premium decile: 1.26 (95% CI: 1.17–1.35), and upper 20% of insurance premium decile: 1.05 (95% CI: 0.99–1.10); (3) 0–5 years old: 1.70 (95% CI: 1.58–1.84), and 6–10 years old: 1.79 (95% CI: 1.66–1.94); (4) residence in metropolitan areas: 1.31 (95% CI: 1.24–1.38) (Figure 3 and Table 2).

### Sensitivity analyses

This showed that the results did not change based on the operational definitions of asthma and ADHD, matching variables, recruitment periods for the asthma group, or matching ratios (all *p*-values < 0.01) (Figure 3).

### Minor research in different age groups

In the case of the 18–60-year-old group, following the same study design, the aHR for ADHD in the asthma group and the non-asthma control group was 1.76 (95% CI: 1.20–2.58). When the experiment was conducted for the entire age group (0–60 years old), the aHR for ADHD in the two groups was 1.21 (95% CI: 1.15–1.27) (Supplementary Figure 2).

### Study 2

Study 2 investigated the reciprocal of the relationship in Study 1, that is, the relationship between ADHD as an independent variable and asthma as a dependent variable. There were 4,132 participants in the ADHD group and the same number in the PS-matched non-ADHD control group. The overall prevalence of ADHD was 41,129/3,474,755 (1.18%) (Supplementary Figure 1). These groups were well-distributed with regard to sex, age, economic status, residential area, atopic dermatitis, allergic rhinitis, and CCI. All SMDs were less than 0.1 indicating that they were well-distributed (Table 3).

**TABLE 3:** Demography of ADHD and non-ADHD groups (Study 2).

Variable	Study (ADHD) group (*n* = 4,132)	Control (non-ADHD) group (*n* = 4,132)	SMD
Sex			<0.01
Female	833	832	
Male	3,299	3,300	
Age, years	10.45 (3.24)	10.45 (3.24)	<0.01
0–5	144	144	
6–10	2,107	2,108	
11–17	1,881	1,880	
Economic status	12.96 (5.89)	12.84 (5.88)	<0.01
<20%	517	517	
20–80%	2,108	2,107	
>80%	1,507	1,508	
Region			<0.01
Rural	911	912	
Metro	3,221	3,220	
Atopic dermatitis			<0.01
Yes	366	365	
No	3,766	3,767	
Allergic rhinitis			<0.01
Yes	242	240	
No	3,890	3,892	
CCI	0.10 (0.32)	0.10 (0.32)	<0.01
0	3,737	3,738	
1	374	374	
2	21	20	

SMD, standardized mean difference and CCI, Charlson comorbidity index.

**FIGURE 4:**
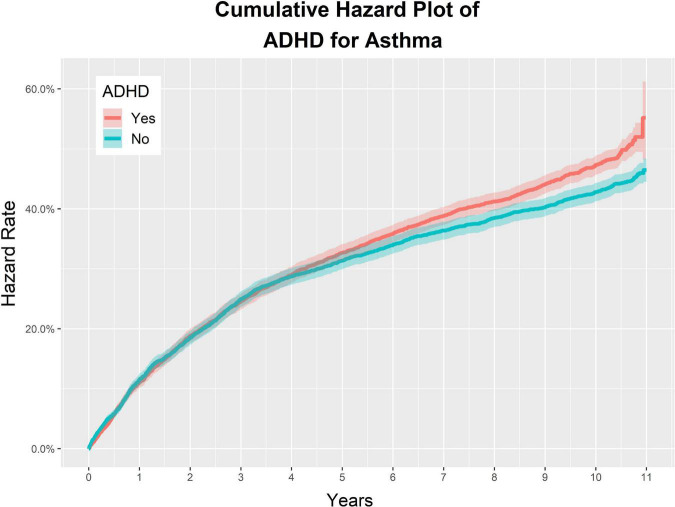
Study 2. Overall cumulative hazard rate for asthma in the ADHD group and the non-ADHD group.

**FIGURE 5:**
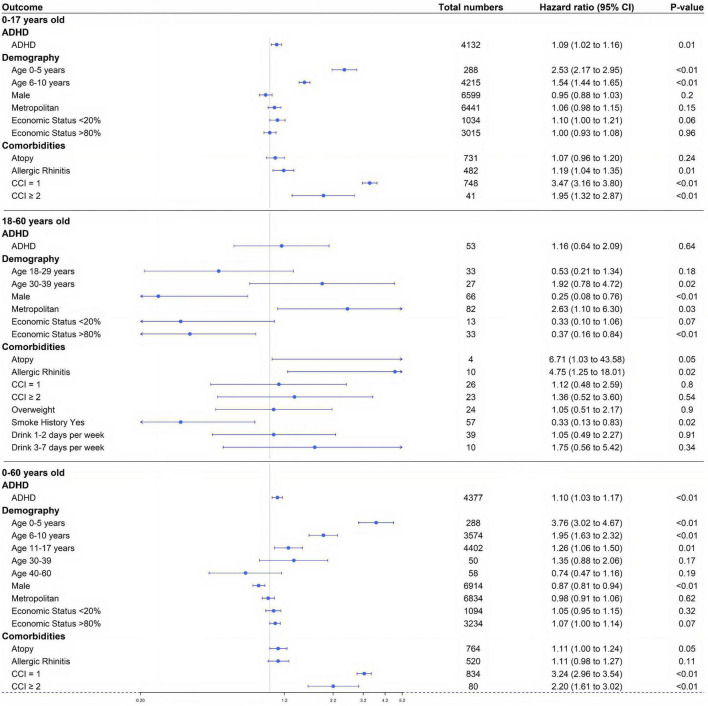
Study 2. Forest plot of adjusted hazard ratio for each factor in a multivariate Cox proportional hazard model for three age groups (0–17, 18–60, and 0–60 years): ADHD, sex, economic status, age, residential area, underlying disease (atopic dermatitis, allergic rhinitis), and Charlson comorbidity index. CCI, Charlson comorbidity index.

The incidence of asthma per 10,000 PY was 68.40 in the ADHD group, and 60.44 in the non-ADHD control group. The aHR of the ADHD experimental group and the control group for asthma was 1.09 (95% CI: 1.02–1.16), indicating that the incidence of asthma in the ADHD group was 1.09 times higher than that in the control group. The univariate crude HR of the two groups for asthma was 1.12 (95% CI: 1.05–1.20) (Figures 4, 5 and Table 4).

**TABLE 4:** Incidence rate and adjusted and unadjusted HRs of each group (Study 2).

Variable	Total	Cases	10,000 PY	Adjusted hazard ratio	Unadjusted hazard ratio
Total	8,264	3,706			
**ADHD**					
No	4,132	1,792	60.44	1	1
Yes	4,132	1,914	68.40	1.09 (1.02–1.16)	1.12 (1.05–1.20)
**Sex**					
Female	1,665	775	65.46	1	1
Male	6,599	2,931	64.01	0.95 (0.88–1.03)	0.98 (0.90–1.06)
**Age, years**					
11–17	3,761	1,455	51.11	1	1
6∼10	4,215	2,062	74.32	1.54 (1.44–1.65)	1.42 (1.33–1.52)
0–5	288	189	133.35	2.53 (2.17–2.95)	2.43 (2.09–2.83)
**Economic status**					
20–80%	4,215	1,877	63.86	1	1
<20%	1,034	495	70.63	1.10 (1.00–1.21)	1.10 (1.00–1.21)
>80%	3,015	1,334	62.83	1.00 (0.93–1.08)	0.99 (0.92–1.06)
**Region**					
Rural	1,823	805	62.12	1	1
Metro	6,441	2,901	64.94	1.06 (0.98–1.15)	1.04 (0.96–1.13)
**Atopic dermatitis**					
No	7,533	3,350	63.56	1	1
Yes	731	356	72.27	1.07 (0.96–1.20)	1.13 (1.01–1.26)
**Allergic rhinitis**					
No	7,782	3,456	63.57	1	1
Yes	482	250	76.57	1.19 (1.04–1.35)	1.18 (1.04–1.35)
**CCI**					
0	7,475	3,128	57.23	1	1
1	748	552	199.70	3.47 (3.16–3.80)	3.25 (2.97–3.56)
2	41	26	123.22	1.95 (1.32–2.87)	2.05 (1.39–3.01)

PY 10,000, per 10,000 person-years and CCI, Charlson comorbidity index.

In the ADHD and matched non-ADHD groups, the characteristics associated with an increase in the incidence of asthma were as follows: patients with allergic rhinitis, CCI = 1, and CCI > 2. The aHRs of the above characteristics were 1.19 (95% CI: 1.04–1.35), 3.47 (95% CI: 3.16–3.80), and 1.95 (95% CI: 1.32–2.87), respectively (Figure 5 and Table 4). The aHR of atopy was not statistically significant, 1.07 (95% CI: 0.96–1.20).

For the demographic factors, aHR values were as follows: (1) male sex: 0.95 (95% CI: 0.88–1.03); (2) lower 20% of insurance premium decile: 1.10 (95% CI: 1.00–1.21), and upper 20% of insurance premium decile: 1.00 (95% CI: 0.93–1.08); (3) 0–5 years old: 2.53 (95% CI: 2.17–2.95), and 6–10 years old: 1.54 (95% CI: 1.44–1.65); (4) residence in metropolitan areas: 1.06 (95% CI: 0.98–1.15) (Figure 5 and Table 4).

### Minor research in different age groups

In the case of 18–60-year-old group, following the same study design, the aHR for asthma in the ADHD group and the non-ADHD control group was 1.16 (95% CI: 0.64–2.09). When the experiment was carried out for the entire age group (0–60 years old), the aHR for asthma in the two groups was 1.10 (95% CI: 1.03–1.17) (Figure 5).

## Discussion

ADHD is a common childhood behavioral disorder that is often diagnosed in children under 10 years of age (25, 26). In Study 1, which investigated the relationship between asthma as an independent variable and ADHD as a dependent variable, the incidence of ADHD was 1.17 times higher in the asthma group than in the non-asthma control group (Figure 3 and Table 2). Among the asthma and non-asthma groups, the aHR for ADHD was higher in 0–5-year-old and 6–10-year-old children [aHR: 1.70 (1.58–1.84) and 1.79 (1.66–1.94), respectively] than in the 11–17-year-old group (Figure 3). In addition, the univariate hazard rate for age in the asthma group was higher in younger children than in the 11–17-year-old group (Supplementary Figure 3).

Other factors that increased the incidence of ADHD included male sex [aHR: 2.83 (2.68–2.99)], living in metropolitan areas [aHR: 1.31 (1.24–1.38)], and the lowest 20% of economic status [aHR: 1.26 (1.17–1.35)]. ADHD is more common in males. Recent research has shown that female patients with ADHD may easily be missed in the diagnostic process if they do not have significant externalizing symptoms (Figure 3) (27). ADHD’s strong relationship with lower socioeconomic status in adults was reflected in the HR value of 1.26 (95% CI, 1.17–1.35) for those individuals with the lowest 20% economic status in the asthma group in Study 1 (28).

Similarly, the greater prevalence of ADHD in metropolitan areas can be explained. In Korea, the difference in population density between metropolitan and rural areas is large, so there is a difference in the density of medical facilities. Therefore, it is considered that the number of times each individual visits a clinic dealing with attention deficit and behavioral disorders to receive medical treatment, especially for diseases such as ADHD, decreases in rural areas, which has resulted in a difference in the incidence of ADHD between these regions. In addition, ADHD has a high incidence in infants and children. Therefore, the larger number of children in cities can affect the difference in prevalence. There is also a higher probability of accessing digital media in metropolitan areas and this increases ADHD-related behaviors as reported in several studies (29).

Study 2 investigated the reciprocal of the relationship in Study 1. The incidence of asthma in the ADHD group was 1.10 times higher than in the non-ADHD control group (Figures 4, 5). This trend was significant in all age groups and was statistically significant in the younger age groups, particularly those younger than 17 years of age, compared to adults (Figure 5). In particular, the HR value for 0–5-year-old children was 2.53 (95% CI: 2.17–2.95), which is the most statistically significant HR value, and the HR for 6–10-year-old children decreased sequentially to 1.54 (95% CI: 1.44–1.65) suggesting that ADHD in childhood is particularly closely associated with asthma.

PS matching can be used to analyze data from randomized controlled trials with larger populations, which has the advantage that bias can be reduced by more effectively treating areas that are more clinically unconstrained. Moreover, studies using PS matching of real registry data often have the benefit of managing datasets and dealing with a broader and less selective population of patients. Our study showed that the distribution of the asthma and non-asthma groups was very similar and that PS matching was well conducted, so the research results are credible (Tables 1, 3). Our research also benefited from the fact that PS matching was conducted on a number of variables such as age, residential area, income level, sex, atopic dermatitis, allergic rhinitis, and other comorbidities included in the CCI. Study results considering the HR of ADHD in the asthma and non-asthma groups through PS matching were consistent with the results of earlier studies and provided statistically robust results (30).

Our study has limitations in that it used data from our Health Insurance Service. Our study could not properly reflect other environmental factors because we only used recorded diagnoses. Additionally, there may be confounders in the environmental factors that might affect ADHD or asthma, such as neglect, abuse, family stress, and air or water pollution. However, because of the limitations of the insurance claim-based data provided by the NHIS, we could not access this information. In addition, even though this study used a large dataset, it only reflects findings in the Korean population and these may not be applicable in other countries. Finally, some inaccurate diagnoses may have been included because of the nature of this retrospective insurance claim-based study.

Despite these limitations, this study has the following intrinsic advantages: (1) it was a large population-based study using national health insurance data, including detailed health records; (2) PS matching was conducted to compensate for the shortcomings of its retrospective nature and regularize comparisons as much as possible; and (3) sensitivity analyses showed the robust nature of the results despite changes in operational definitions, matching variables, recruitment periods, and matching ratios.

## Conclusion

In conclusion, our study with long-term follow-up has shown that the incidence of ADHD in patients with asthma was 1.17 times higher than that in patients without asthma, and the incidence of asthma in patients with ADHD was 1.10 times higher than that in patients without ADHD. Clinicians should pay attention to whether patients with asthma, especially male patients and children, exhibit symptoms of ADHD. We suggest follow-up studies to compare the prevalence of ADHD according to asthma severity to verify specific associations. In addition, we showed that younger patients with ADHD are at a higher risk of asthma and need to be monitored carefully to observe and manage any symptoms of asthma. Taken together, asthma and ADHD have a bidirectional relationship; therefore, childhood asthma and ADHD should be rigorously managed.

## Data availability statement

The raw data supporting the conclusions of this article will be made available by the authors, without undue reservation.

## Ethics statement

This study protocol was reviewed and approved by the Institutional Review Board of JBNUH (IRB No. CUH 2021-10-031). As information used for analyses from the NHIS-National Sample Cohort database was encrypted and anonymized, informed consent was not required.

## Author contributions

JK: conceptualization, supervision, validation, visualization, and writing—review and editing. SY, MK, and ML: data curation and formal analysis. HP and JK: investigation, methodology, and project administration. JK, SY, MK, and ML: resources and software. HP, YK, DN, SJ, JK, J-HL, and YY: writing—original draft preparation. All authors contributed to the article and approved the submitted version.

## Supplementary material

Supplementary Figure 1 Study 2. Overall distribution of ADHD patients binned by age.

Supplementary Figure 2 Study 1. Forest plot of adjusted hazard ratio for each factor in a multivariate Cox proportional hazard model for three age groups (0–17, 18–60, and 0–60 years): asthma, sex, economic status, age, residential area, underlying disease (atopic dermatitis, allergic rhinitis), and Charlson comorbidity index. CCI, Charlson comorbidity index.

Supplementary Figure 3 Study 1. Subgroup analysis of the asthma group; univariate cumulative hazard rate of age, sex, region, and economic status in the asthma group.
