# Supplementary figures and images for: Long-term bidirectional association between asthma and attention deficit hyperactivity disorder: A big data cohort study
Source: Front Psychiatry. 2023 Jan 19;13:1044742. doi: 10.3389/fpsyt.2022.1044742 (PMC9893024; doi:10.3389/fpsyt.2022.1044742)

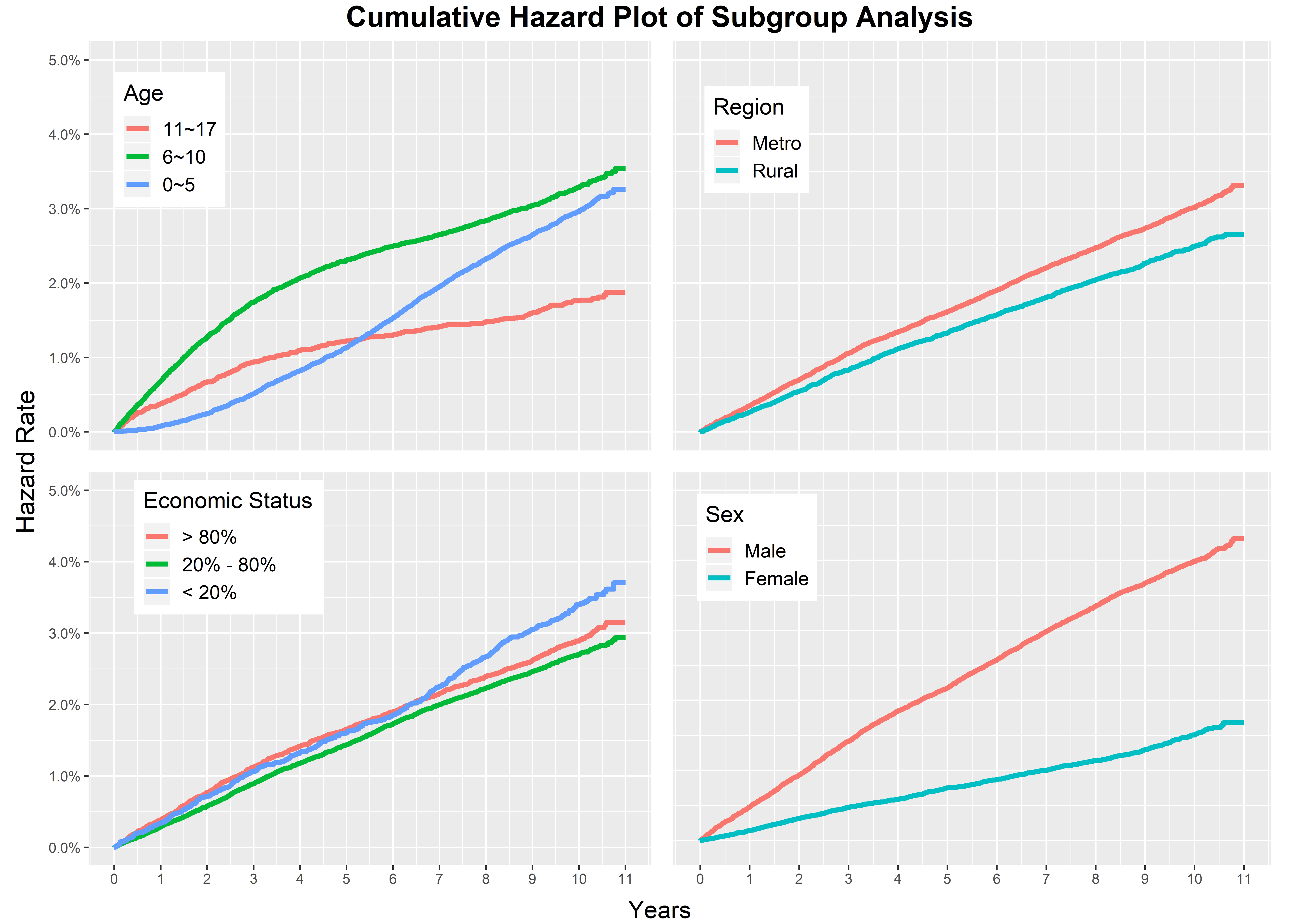

Supplement: Supplementary Figure 3 — Study 1. Subgroup analysis of the asthma group; univariate cumulative hazard rate of age, sex, region, and economic status in the asthma group. [file Image_3.JPEG]
